# Clinicians’ experiences of obtaining informed consent for research and treatment: a nested qualitative study from Pakistan

**DOI:** 10.1186/s12910-024-01119-8

**Published:** 2024-11-15

**Authors:** Rakhshi Memon, Muqaddas Asif, Bushra Ali Shah, Tayyeba Kiran, Ameer B Khoso, Sehrish Tofique, Jahanara Miah, Ayesha Ahmad, Imran Chaudhry, Nasim Chaudhry, Nusrat Husain, Sarah J L Edwards

**Affiliations:** 1 University College London, London, UK; 2 Pakistan Institute of Living and Learning, Karachi, Pakistan; 3 University of Manchester, Manchester, UK; 4 St George’s University of London, London, UK; 5 Ziauddin University Hospital, Karachi, Pakistan; 6 Mersey Care NHS Foundation Trust, Liverpool, UK

**Keywords:** Young people, Informed consent, Shared decision making, Randomised controlled trials, Autonomy cultural differences

## Abstract

**Background:**

Informed consent is considered to be the standard method for respecting the autonomy of individual participants in research and practices and is thought to be based on several conditions: (1) providing information on the purpose of the research or a specific treatment, what it will entail, (2) the participants being mentally competent to understand the information and weigh it in the balance, and (3) the participants to be free from coercion. While there are studies of informed consent in other countries, especially Low and Middle Income Countries (LMICs), this study explored the experiences of clinicians regarding the process of obtaining informed consent to participate in a Randomised Controlled Trial (RCT) in particular and treatment in general in healthcare settings, both general and mental health, specifically focusing on the tension between individualistic concept of autonomy and collectivist values in cultures such as Pakistan.

**Methods:**

Qualitative interviews with 20 clinicians from healthcare settings in Pakistan who also served as recruiters in a suicide prevention RCT in Pakistan. The interviews were guided by semi-structured topic guide. All interviews were audio-recorded and transcribed verbatim.

**Results:**

The interviews revealed that shared decision making was more morally important than individual autonomy, the role of the family played a dominant part in the consent-taking procedure, the decision of the elder and/or family patriarch took prominence, and that clinician-researchers encountered significant challenges in consent process in Pakistan, while recruiting patients into the trial as well as during routine treatment processes in healthcare settings. Four distinct themes emerged which were (1) Family deciding for patients, (2) Benefits of involving family in consent process, (3) Gender disparity in consent process, (4) Challenges experienced by clinician-researchers during consent process in Pakistan.

**Conclusions:**

The concept of consent is generally considered important in many cultures, however, there are two strands of understanding. There seems to be consensus that participant agreement is necessary to protect the participant but with regards to autonomy there are significant cultural differences whether it is the right for autonomy of the individual (individualistic concept) or family, community, or expert authority in other cultures. In Pakistan clinician-researchers sometimes preferred one approach and sometimes the other as they appreciated the interests of the patient to be.

## Background

In healthcare settings, obtaining informed consent for medical and surgical procedures involves a process where communication between the clinician and the patient leads to the patient’s explicit authorisation to undergo a specific medical intervention [1]. For the authorisation to be valid, the patient must have the capacity to consent, engage in discussions to understand all pertinent information, provide consent voluntarily, and effectively communicate their decision [2]. Informed Consent is also an underpinning condition for safeguarding the autonomy of patients/participants in Randomised Control Trials (RCTs). According to Hey and Troug [3], if a participant fully understands the risks for taking part in the trial and gives informed consent then it is ethically justifiable to conduct the RCT. The recruitment of mentally competent human subjects in clinical trials without their consent is generally thought to be unfair and exploitative except in certain circumstances such as emergencies [4].

Informed consent process is considered as valid if it includes information disclosure, voluntary choice, and decision-making capacity [5]. Disclosure of relevant information includes four key elements: understanding the risks, benefits, alternatives, and general knowledge associated with the medical procedure or intervention in question [6, 7]. However, ensuring adequate understanding is challenging for vulnerable groups including those with limited education or health literacy, or those who experience mental health issues [8, 9].

Informed consent guidelines internationally, emphasize the importance of specific information needs of potential participants and the ways of providing this information [10]. However, the actual practices of informed consent can vary by country and may be influenced by cultural contexts [11–13]. In recent years, there has been a noticeable trend towards adopting more patient-centred informed consent standards, reflecting a shift toward prioritising the patient’s involvement and understanding in the decision-making process [14].

Health research activity has increased significantly in low- and middle-income countries (LMICs), and research has highlighted issues around informed consent [15–19]. Obtaining informed consent in LMICs using traditional paper-based consent forms is challenging due to factors such as high illiteracy, lack of transparency, and use of scientific jargon [19]. In low resource settings, decision to participate in a trial may be considered as an opportunity to secure access to healthcare services. Poverty may also lead to ‘competition’ to access the research-related benefits, with a risk of disturbance at household or societal level [20]. Moreover, in LMICs it is important to understand the role of local values and structures that may impact individual’s capability to consent to a study [21].

Family plays an important role in consent process in some cultures such as Asia, where man of the house makes all the decision for the family, this may make other members, women in particular, a vulnerable group in consent process leading to unwilling participation or undue exclusion [22]. In addition, doctors receive huge respect from communities, particularly in the rural areas which are physician deficient, and their role is accepted as a principal decision-maker in clinical situations [23], which is an important fact to be considered while carrying out research activities in healthcare settings. This has implications for informed consent process [22, 24]. Al-Saadoon and Al-Adawi [25] concur that application of the individualistic model of informed consent tends to marginalise cultural and social factors of traditional societies. According to Tham et al. [26], the process of informed consent should be customised in keeping with literacy and cultural context of participants in a trial. In some low income settings such as India, hierarchical relationship between potential research participants and physicians caused disruption in the sharing process leading to patients’ failure to share their concerns or emotions before making a decision regarding participation [27].

In Pakistan, to understand experience of consent process for participation in suicide prevention RCTs, it is important to understand the healthcare context. The recruitment sites to approach and identify potential research participants for such trials are emergency and medical units where these patients are presented after self-harm attempt and treated [28]. It is important to note that clinicians in Pakistan are mostly involved in academia and practice and less involved in research [29]. A code of ethics for all the medical practitioners including clinician-researchers has been formulated by the Pakistan Medical and Dental Council (PMDC) [30]. However, cultural values in Pakistan offer a challenge to the practice of healthcare and research ethics in Pakistan. Healthcare decision making in Pakistan is done by family or physicians [31]. Patient awareness of their rights to informed consent and privacy is often limited and there is absence of implementation of standard informed consent procedures [32].

While the concept of consent is generally considered important in many cultures, it is essential to recognise that the specific understanding and significance of consent can vary across cultures [33]. There are two strands of understanding. There seems to be consensus that patient agreement to participate in clinical research and/or a treatment process is necessary to protect the patient’s interests, but with regards to autonomy, there are significant cultural differences whether it is the right for autonomy of the individual (individualistic) or family, community, or expert authority in other cultures. The fundamental difference between the individualistic political philosophy orginating in some Western countries and other contemporary healthcare settings in other political and cultural countries must be acknowledged [34, 35]. The individualistic concept of informed consent is to respect the human dignity and protect autonomy of individuals [36]. Personal autonomy is defined as self-rule that protects meaningful choice and is free from controlling interference and limitations [37, 38]. The international concept of informed consent emphasizes the rights of an individuals to receive adequate and appropriate information on their autonomous decisions while taking part in a study. In the context of health research, Individuals have the right to exercise their autonomy in the context of healthcare research, and if this is diminished or impaired, they need to be protected from harm and abuse [37].

This study explored the experiences and views of clinician-researchers around the intricate process of obtaining informed consent in the context of a suicide prevention RCT, with a particular emphasis on the juxtaposition between individualistic ideals of individual autonomy and the prevalent collectivist values observed in cultures like Pakistan, where shared decision-making is deeply ingrained as a norm.

## Methods

### Design

A qualitative study nested within a Randomised Controlled Trial (RCT) on suicide prevention in adolescents in Pakistan [39]. This RCT aimed to recruit young self-harm survivors from emergency and medical settings from across Pakistan.

### Setting

Clinicians (who served as trial recruiters) were recruited from health facilities from three participating cities of the RCT in Pakistan. There were five participants from Lahore, six from Rawalpindi and nine from Karachi. These were psychiatrists, General Practitioners (GPs) and Emergency Care doctors. These healthcare facilities were the recruitment centres for suicide prevention trial [39].

### Participants

Convenience sampling was used, and participants (*n* = 20) were recruited depending on their availability and willingness to participate in an interview.

### Inclusion criteria

Medical doctors working at healthcare facilities involved in recruitment of participants in a suicide prevention RCT from Karachi, Lahore and Rawalpindi, Pakistan. These medical doctors were either directly engaged with the research team recruiting adolescent self-harm survivors for an RCT or supporting research team to approach and identify potential participants.

### Exclusion criteria

Medical doctors and other health professionals not recruiting participants in RCTs from Karachi, Lahore and Rawalpindi, Pakistan.

### Procedure

Potential participants received information sheets upon invitation and had questions addressed by the research team. Informed written consent was obtained. Researchers conducted semi-structured interviews using an approved topic guide [40] by qualitative research experts (RM and TK) to comprehend experiences of clinicians as trial recruiters regarding process of taking informed consent from potential trial participants to be enrolled in a suicide prevention trial [41]. The topic guide was also discussed with wider stakeholders including clinicians. The participants were invited to respond in Urdu, English or Urdu/English mixed - whichever language they felt comfortable in. The purpose of the semi-structured interviews, using a topic guide was to understand clinicians’ experiences while obtaining informed consent and to examine whether the role of the family had a part to play when enrolling patients to the trial. Ethics approval for the main study was obtained from the National Bioethics Committee of Pakistan (Ref: No.4–87/NBC419/19/1213) and the Research Ethics Committee University of Manchester (Ref: 2019-5024-10755). Ethics approval for RM’s thesis was granted by Science and Technology Department, University College London (Ref. STSEth159). Interviews were conducted from February 2020 till December 2020. Each interview was digitally audio recorded and lasted between 50 and 60 min.

### Analysis

Data were analysed using Braun and Clarke’s reflexive thematic analysis approach [42]. In the initial familiarisation stage, researchers read and re-read the transcripts to fully immerse themselves in the data. After this stage, a line-by-line coding process was conducted. A draft framework was developed in which themes were identified from all interviews. The draft theoretical framework was applied systematically to the whole dataset during indexing. Here the data from transcripts were copied and pasted alongside the relevant themes that were listed in the draft framework. Subsequently, a comprehensive comparison between the data and themes took place, leading to the refinement of the analysis framework. Data were summarised into tables developed using MS Word software for each theme listed in the analysis framework, during the charting process. Tables were reviewed during the final stages, i.e., the mapping and interpretation phase. This enabled all key ideas and the data to be compared and discussed within the whole research team, and to identify the final framework that synthesised and interpreted the data as a whole.

## Results

Eight participants were female and 12 were male. The age group of the participants ranged from 31 years to 64 years. All participants were from urban areas practising in Karachi, Lahore and Rawalpindi. These are the three main cities of Pakistan.

Four main themes emerged from the clinicians’ interviews.

1. Family deciding for patients.
2. Benefits of involving family in consent process.
3. Gender disparity in consent process.
4. Challenges experienced by clinicians during consent process in Pakistan.

The interviews revealed that in the cultural context of Pakistan, shared decision-making in clinical settings superseded the concept of individual autonomy. This can cause moral dilemma for the clinician as the jurisdiction under the Hippocratic oath requires them to uphold their individual patient’s right to autonomy. However, they also understand that in the majority of cases, it is a cultural fact that family involvement is for positive reasons and for the welfare of the patient. Participants talked about potential role of families in decision-making related to participation in research and treatment.

### 1: family deciding for patients

Families play an important role in healthcare research decision making in Pakistan. According to one respondent,

“*So*,* when you are conducting research here*,* you have to keep in mind that the family would be involved*,* it is a cultural thing mostly. Sometimes it’s irritating because we must wait for particular family member to show up to make the decision for the family or the patient but then you also have to understand …. that’s how we are here I mean it’s not just the patient making the decision for themselves*,* it’s everybody involved so while practicing here we have to keep that in mind.*” (RWP Int-02 line 115–119).

One respondent stated similar challenge in treatment context, *“*,* many times*,* family is the decision maker and not the patient. In most instances*,* the family would decide if the patient should be admitted or not*,* or whether to request the doctor to discharge the patient etc. So*,* in most cases it is the family who decides.”* The same respondent expressed their irritation with regards to the role of the family at the time of taking consent from their patient to participate in the research,

*“Overprotective or intrusive. They are intruders.”* (Lhr Int-03 line no 28–29).

Most clinicians stated that the reason for the family or elders taking over the decision making was, from the notion of protecting the patient from getting anxious when receiving bad news. As one clinician said,

*“So*,* one should consider whether you are with the patient or with the family. If you do not tell the patient because of the family*,* it means that you are acting against the patient in favour of the family. Mostly in emergency this happens. If we ask the family*,* why do you not want to tell the patient? They would probably reply that he will die from anxiety and stress. The family does not prepare the patient. They try to handle in their own way. Basically*,* the patient is just a dummy in this situation and family is taking all the decisions. This is a difficult situation for the doctor.”* (Lhr Int-01, line no 430–436).

Another said,

*“We will ask about the family consent about any intervention we are going to perform on the patient (as part of the research)*,* be it their parents or brother or guardian anyone who will be the immediate guardian of that patient.”* (RWP Int-01, line no 123–125).

One respondent disclosed dire consequences of absence of patient’s consent in treatment process and hence ignorance of what will be happening to them,

*“I have seen patients whose arms get amputated due to the consent of their family members*,* but the patient doesn’t know that this is going to happen to them. So that is what should not happen basically in my opinion. The doctor should have talked to the patient*,* the patient himself should at least understand the basics. At least patient should understand what is going to happen to them*,* take the final decision on consent as given by the family members*,* the male figures in their family. The patient should know what is going to happen to them that is the minimum what can be done at least.” (RWP Int-04*,* Line 111 to 117)*.

Participants also talked about vital role of family in consent process for research, they stated,

*“And sometimes when we recruit patients directly from hospital then we have to tell their families too; sometimes patients give us consent*,* but families do not agree and so there are numerous issues we face in RCTs.”* (Khi Int-04, line no 208–210).

They went on to suggest, *“Also as I told you before that in Pakistan*,* families have hold over an individual`s decision as well as on the signing of the consent form (for participation in research).”* (Khi Int-04 line no 219–220) and they reiterated the influence of families on the area of consent,

*“Some issues around consent; families are very influential; patient agrees but family say no we don’t want to involve our patient in this trial.”* (Khi Int-04 line no 382–383).

Another respondent alluded,

*“In our culture patient does not come alone rather*,* he comes along with the family. Family then asks us that we cannot come again and again or exchanging telephone number so problem arises at this stage and in prospective studies we face problem on follow-up in our Pakistani culture.”* (Khi Int-05 line no 73–76).

### 2: benefits of involving family in the consent process

Some clinicians recognised that family involvement can help with compliance. One stating, *“In the cultural context of Pakistan we need to allow patient attendant to sit in the session (during trial intervention) to accompany a female patient if she is brought by the son or sister-in-law; then compliance will be good but if you ask a female patient to take the session alone then they won’t show good compliance.*” (Lhr Int-02 line no 100–102).

Participants also talked about importance of availability of family and their consent in situations where it is not possible practically to explain the process of treatment or intervention to the patients due to a variety of factors including patient’s mental state or if the patient is not medically fit. One participant said, “*Well to be honest*,* sometimes*,* you cannot rely on the patient consent when the patient is not medically fit to give consent and you have to consult family as they are there. For example*,* when we want to discharge the patient or provide them ECT*,* we need consent to that*,* so we do not ask the patient*,* we mostly don’t because patients do not listen*,* they are not in a stable state of mind. It’s mostly the family members around or some particular person who is responsible for making decision for the family and we have to involve them”.* (RWP. Int-02-LINE 105–111)

Another respondent said,

*“Joint family system is very common here so if they are bringing a female patient on daily basis or even three times a week then it could disrupt the whole family and there will be questions as to why she is going? where is she going? What sort of issues has she been dealing with? What is the problem?”* (Lhr Int-02 line no 142–146). This respondent went on to reiterate the importance of family involvement in the consent taking process during research as the responsibility to bring patient to the healthcare facilities lies with the family,

*“Obviously consent issues will arise as the family is bringing the patient*,* so family consent will have to be taken as well as from the patient. Family consent is very important*,* if consent is only taken from the patient*,* then it won’t be reliable it is necessary to take the consent from the family.”* (Lhr Int-02 line no 157–159).

There was recognition that family set up is very intertwined and that family is very much involved in the care of the patient. According to this respondent,

Mostly it is family consent but sometimes if the patient is not willing to reveal certain kind of information, then we try to interview the patient alone. We take consent from the patient first and then from the family. The family has to be involved, also.

According to one respondent, there are positives and negatives of the involvement of family, they alluded,

*“What usually happens here is we will take the consent from the patient*,* but we are aware that the decision maker is only the family. We know that the consent we are taking from patient is family informed. Patient who has insight will discuss that they are getting negative thoughts*,* and they know that they should be involving the family. However*,* some families are very intrusive*,* and they do not let the patient speak. You can see that the patient is sitting there and wanting to speak but family members keep telling their version and keep talking. Even when we ask the family to leave so we can interview the patient on their own. The family members keep looking in again and again (laughing) to describe the patient’s symptoms. So*,* it is really difficult to deal with such families. It becomes difficult. Although*,* some families are really helpful*,* and their involvement is a great help. But mostly their intrusion is irritating and makes things worse for the patient. Everyone has his/her own opinion according to age gender*,* experience etc. which may be different to the patient’s opinion.”* (Lhr Int-01 line no 412–429).

### 3: gender disparity in consent process

Most clinicians accepted that family involvement and decision making was part and parcel of the Pakistani culture but the situation is different based on the gender of the patients. Clinicians highlighted that men generally have the autonomy to make decisions for themselves;

When it comes to male patients, it (consent) is not a problem because they decide for themselves. I don’t have any experience with transgenders so I can’t comment on that but male patients usually decide for themselves. They may need some time to discuss it with family but it is not a problem especially for men above 18. (RWP Int-04, Line no 421 to 425)

Participants stated that family involvement in healthcare related decision making was more so in the case of female patients. *“I think in terms of family involvement in our culture*,* yes families are heavily involved even in people who have reached adulthood and specially if we look at women. They are not generally the decision-maker in terms of their lives even if they say they are. Even when they have reached the age of 50*,* borne four children. So*,* when we are working with women*,* we always involve their husbands or mother-in-law otherwise it’s very difficult for them to continue to remain engaged because there are a lot of hurdles that come in their way if families are not supportive*” (Khi Int-08 line 170–181).

Clinicians were also concerned about the dignity and privacy for their female patients due to various reasons such as biological differences, cultural norms and sensitivity. One respondent expressed, “*sometimes patients come to us from KPK*,* like Peshawar and Quetta but husbands do not come along. Females are accompanied by their fathers-in-law or brothers-in-laws and during history taking*,* we have some problems; we need to share certain information with husbands; but we have to share with fathers-in-laws or brothers-in-laws or sometimes they come with their siblings. So*,* we face difficulties in how to prepare the patient for treatment”*.

Group conformity matters to the point that in rural areas if a female wants tubal ligation, the husband needs to give consent. According to another clinician, *“I mean it’s her body and her choice*,* but you know husband and if husband wants to have a vasectomy*,* does the wife gives consent? That is unthought of. So*,* you have built-in hierarchy like grandfather*,* son and grandson dictates that this is the person you are supposed to marry*,* this is what they are supposed to have*,* in this line you should go etc. So*,* consent is quite different here and those of us who work here realise that one needs to talk to the relevant individuals*,* the decision makers before taking actual consent so it is quite different from what we perceive in the west.”* (Khi Int-01 line no 305–315).

One clinician stated, “*I am talking about lower socioeconomic or may be middle class. What they do is they over assert in the sense*,* for example*,* a lady who needs to get family planning done*,* she is not allowed or for that matter*,* she has to get permission or signed by the husband to save their own I would say reputation. The clinicians*,* when they are installing some kind of family planning device*,* they ask you need to bring your husband and he has to sign the permission letter and then only we will install the device” (Khi Int-05 line no 300–305).*

They went on to express their perplexity on female dependency on their male family members to be the decision makers on their behalf,

*“I am still confused as to how we would handle this in female patients even if we explain the whole thing to the patient*,* I have seen it multiple times that they still refer me to their father or to their husband to take the decision and I have tried it*,* I have tried it many times to explain it to them that you are the one who need to decide what is going to happen to you and she agrees with me at the time but then she again looks at her husband for the consent. It is something which is deeply rooted*,* and I don’t know if we can expect one study or one thing to change that*,* I don’t know if that is possible.”* (RWP Int-04 line no 391–397).

One respondent talking about practising in the community setting stated,

*“In the community*,* in those urban areas*,* people are of low socioeconomic status*,* right. So*,* in that case even if ‘bahu’ (daughter-in-law) comes over to get her pregnancy test done or confirmation test done*,* in this case*,* sometimes you must have a rapport built to be able to ask the mother-in-law that can I please talk to your daughter in law and if you can wait outside? (‘mai zara sa unko deakh lu*,* aghr app bahir intezar ker ly’). You know*,* it is important how I deal with the mother-in-law*,* and when I send her out then I can quickly ask whatever I need to from the female from the confidentiality point of view but other than that it is very difficult ”* (Khi Int-06 line no 262–279).

### 4: challenges experienced by clinicians during consent process in Pakistan

Participants talked about moral distress caused to clinicians due to undue interference of family while managing patients, they stated *“It’s tough*,* very tough. Somebody you want to enrol and their husband or their chaperone sort of refuses to bring them to and from the clinic. They don’t want to do that*,* and you know that this is a genuine client who genuinely needs the therapy. Hmm*,* it engenders stress*,* tension as we are not able to practice as we would like to*,* but you get attuned to that*,* and you work through this lack of understanding I would say.”* (Khi Int-01 line no 319–327).

They said that distress is even more so when they have to involve other relatives as well who does not belong to the immediate family. Clinicians expressed their frustration when decision takers may not be directly related to the patient. According to one clinician, *“unfortunately*,* in our country what happens is that we take consent from parents and paternal*,* maternal uncles they have no relation*,* no direct relation to the patient but we have to take consent from them and that is considered ok. Personally*,* I don’t agree that it should be how it happens here*.” (RWP Int-04 line no 59 to 62).

One respondent commented on issues of preserving confidentiality, *“families want an exact verbatim account of what conversation you had with their son or daughter (during their participation in psychological interventions).”* (Khi Int-07 line no 349–351). They went on to state,

*“What happens is that they start getting upset and say I have brought my son or brother to you*,* and I have paid fees to you so why are you not going to tell me*.” (Khi Int-07 line no 351–353).

The same respondent said,

*“If one takes the time to make the family understand; they will understand but some are very stubborn. At least*,* half a dozen times I have had to walk from the patient’s bed side when the brother or somebody has said No Psychiatrist!……….Majority of the times (patient is wanting you but the family is not letting you treat them) and this is challenging! The poor patients are desperate on the other hand.”* (Khi Int-07 line no 412).

Participants also talked about disturbance caused by the delays in treatment when they have to wait for family members to come and provide consent as patients refuse to make decisions on their own. One clinician mentioned, “*sometimes it’s irritating when we have to wait for a particular family member to show up to make the decision for the family or the patient*,* this delays the process of treatment but then we also have to understand the limitations that how things are here and while practicing here we have to keep that in mind.* (RWP Int-02-LINE 115–119)

The conflict among the family members over the choice of treatment also cause challenges for clinicians during treatment process. Patient’s consent does not matter here for the family and the one who is more influential makes the decision regardless of the consequences. *“Sometimes patient wants to do one thing and the family members want the other thing or sometimes different family members have different choices. We had a patient who wanted to get ECT but his mother was not willing*,* the father was willing. So you were in a fix. We tried to explain but they left before ECT as the mother was not happy and convinced and they left the hospital”.* (RWP-Int-02-LINE 128–134)

Participants also highlighted that while recruiting patients for research, the literacy level of the potential participants may cause a challenge in understanding what the research is all about. *“Aa to be honest*,* most of the patients who come here. They’re not that much educated. They are ahh*,* their literacy rate is*,* their literacy level is not that much that I can explain to them what the YCMAP (suicide prevention intervention for young people) is before taking informed consent”.* (Khr-Int1, Line 24–26)

*“Actually first of all we have discussed earlier that culture*,* norms*,* values and education status and understanding these are the barriers in consent process that should be addressed in conducting a study”.* (RWP-Int 03-Line 176–177)

## Discussion

This qualitative study illuminated common ethical challenges during recruitment of participants in intervention related research experienced by clinicians who recruited participants into a suicide prevention trial in low-income settings and informed consent for treatment in general. Our findings indicate significant gaps in the implementation of the informed consent process in collectivist societies. The family role in gaining consent and in care management and treatment planning of the patient remains central in the Pakistani context and interconnectedness superseded individual autonomy and decision making. Although in Pakistan, the legal age for consent to various aspects, primarily related to personal decisions and actions is 18 years, clinicians stated that to get engagement from the patient for successful recruitment in the trials and treatment, it is equally important to effectively explain the purpose of the trial and treatment to the family. Trust of the family varies from person to person and on the socio-economic status. If the family was educated, it seemed easier to develop trust and explain the concept of confidentiality and informed consent. On the other hand, in rural communities where there may be lack of awareness and due to cultural trends particularly gender stereotypes, family decision dominates, and the family has the last word.

Gender inequality around decision making and dependence on family and other male members of the family came up repeatedly. Analysis of the views deduces that clinicians find this societal constraint on female patients unjust, and this causes them moral distress. When conducting clinical research in Pakistan, it is crucial to accept that the family would have to be informed at each and every step of the process and in particular, when working with women, their husbands or mother-in-law must be involved for them to remain engaged. A common aspect discussed in the interviews was that we need to take into account the family’s role in the consent procedure. It is crucial to recognise that in collectivist cultures, lack of family involvement may in fact be detrimental to participant engagement. Close alliance with family members may facilitate the informed consent process and also help build general awareness about research in the wider community.

The results clearly show that the role of the family in decision making is largely considered as an enabler by clinicians and researchers rather than a barrier. Most clinicians acknowledged that the family support system provides great advantages with compliance and management of the treatment in the patient’s own interests. The pre-eminence of collective decision making in Pakistani families was emphasised. Due to cultural and religious boundaries, building rapport with the family and explaining and talking to them does not only facilitate the consent process but also promotes awareness. Acting in accordance with decisions taken by elder members of the family is regarded as a sign of respect and the influence of elders is considered as a blessing by the young. The fundamental difference between the liberal political philosophies and health systems in countries with other political and cultural systems must be acknowledged. Individual consent may be regarded as less morally important than it is in liberal contexts with families making collective or patriarchal decisions. It becomes an ethical imperative to examine, as to what extent should individual consent be instituted by researchers in the cultural, social and religious context of Pakistan. A study carried out by Fatima [43] in India, Iran and Pakistan revealed challenges to gaining informed consent such as illiteracy, language barriers, cultural influences, religious influences, and false perceptions which impacts recruitment rates too. Ekmekci and Arda [34] report that a number of authors find fault with western model of giving too much importance to individual decision-making and recommend involving ‘others’ in the informed consent process. Their rationale is that an individual is influenced by the family and community all through their life and involving others provides the individual the confidence and support at the time of decision making. There seems to be consensus that patient agreement is necessary to protect the patient [44–46] but with regards to autonomy there are significant cultural differences whether it is the right for autonomy of the individual (largely orginating in the west) or family, community, or expert authority in other cultures.

The Western clinical research rules are often very prescriptive and emphasise consent by the individual patient and duties for the profession in respecting that consent. These can create grounds for ‘medico-cultural’ conflict if transposed on other cultures with different religious and philosophical beliefs and traditions [47]. Johnson et al. [47] research into Asian medical traditions (Ayurveda and Traditional Chinese Medicine) reveal that although ethics was mainly concerned with principles of behaviour for the profession, these ethical precepts were underpinned by humaneness and compassion. Codes of ethics are necessary to protect vulnerable research subjects. It is important to remain mindful in the moral interpretation of rules that whether what is required to be done to participants is in reality something we would consider to be done to us or our loved ones. Arthur Kleinman, psychiatrist, and anthropologist argues that in developing countries clinical research must be contextualised within the everyday beliefs, values and power dynamics [48]. According to Kleinman, it is not the individual who makes the choice in isolation but as part of a network of relations. The concept of relational autonomy has been discussed by other authors especially feminist scholars within liberal societies [49–51] in other theoretical contexts as well such as in the context of vulnerability [52], paternalism [53] and maternalism [54]. The concept has been discussed in informed consent in Pakistani medical settings [23, 55, 56]. Evidence also highlighted the potential positive impact of relational autonomy in clinical practice and research [57]. Often unrecognised by western clinical researchers, the differences in conceptions and expectations and norms can create conflict between cultural differences when conducting research. As more and more western medical research is being done in non- western settings to ‘decolonise’ or bridge research gaps, it becomes an ethical imperative to study the local impact and perceptions of this exogenous interaction with the indigenous medical research practices and norms [23, 56, 58].

Given that research is intended to ultimately improve overall outcomes for these participants, it may be beneficial to tailor the informed consent process to their cultural nuances. Respecting diverse values and beliefs while taking steps to sensitise participants and their families to the research process [59], could be useful mechanisms to eliminate the aforementioned ethical barriers. Our emerging themes delineate important findings that must be considered to design an informed consent process that meets the needs of the participants whom the research is intended to serve, within the contextual framework of their cultural, religious, and moral belief systems.

The study focuses the encounter between individualistic autonomy and collectivist values in informed consent. It is an important addition to investigations on cultural norms’ role in RCT consent in low-resource countries like Pakistan. This complements understandings on ethical universality in diverse settings. While specific to Pakistan, practical collectivist values may echo in similar contexts, not universally.

### Limitations

The process of informed consent was from the perspectives of clinicians only. The perspectives of other researchers (psychologists) who were involved in recruitment in this suicide prevention trial and trial participants themselves were not explored. Including the voices of individuals involved including participants (adolescents) and their families (parents/guardians in particular) in the informed consent process would provide a more comprehensive understanding of how it is perceived or understood. In additions, the study did not explore the experiences and views of clinicians on obtaining informed consent from parents/guardians of participants (adolescents). This would have provided insights on cultural aspects and challenges of obtaining informed consent from parents to recruit young people into clinical trials and treatment process for this population. The number of clinicians in the qualitative study was small and mainly recruited from three centres of a single mental health RCT reducing its generalisibility.

## Conclusion

In LMICs such as Pakistan, the significance of collective consent is rooted in communal values and traditions where decision-making often involves family and community members. The belief behind this collective consent is that decisions are not individualised rather they contribute to the overall wellbeing of the community. The wisdom and experiences of the elders and community leaders guide the decision-making process and reflect historical and cultural significance, a sense of interconnectedness, foster a sense of shared responsibility and reinforce best interests as a holistic approach while prioritising the welfare of the community over individual autonomy. Often unrecognised by western clinical researchers, the differences in conceptions and expectations and norms can create conflict. As more and more western medical research is being done in non- western settings, it becomes ethically exigent to study the local impact and perceptions of this exogenous interaction with the indigenous medical research practices. The revised version of the Declaration of Helsinki in acknowledgment of cultural differences has also stated that the informed consent process should not only be concerned with individual consent but should also take into account family and in some circumstances may also include community leaders. Thereupon, it is crucial to be mindful when conducting clinical research in low resourced countries, that it is contextualised within the everyday beliefs, values, power dynamics and moral interpretation of cultural norms.

## Data Availability

Data is provided within the manuscript.

## Abbreviations

WMA: World Medical Association
RCTs: Randomised Controlled Trials
LMICs: Low and middle income countries
HICs: High Income Countries
GPs: General Practitioners
